# A CMR study of the effects of tissue edema and necrosis on left ventricular dyssynchrony in acute myocardial infarction: implications for cardiac resynchronization therapy

**DOI:** 10.1186/1532-429X-14-47

**Published:** 2012-07-17

**Authors:** Robert Manka, Sebastian Kozerke, Andrea K Rutz, Christian T Stoeck, Peter Boesiger, Juerg Schwitter

**Affiliations:** 1 Institute for Biomedical Engineering, University and ETH Zurich, Zurichm, Switzerland; 2 Department of Cardiology, University Hospital, Zurich, Switzerland; 3 Centre Hospitalier Universitaire Vaudois, CHUV, Rue du Burgon 21, Lausanne, 1011, Switzerland

**Keywords:** Cardiovascular magnetic resonance, Tagging, Myocardial infarction, Dyssynchrony

## Abstract

**Background:**

In acute myocardial infarction (AMI), both tissue necrosis and edema are present and both might be implicated in the development of intraventricular dyssynchrony. However, their relative contribution to transient dyssynchrony is not known. Cardiovascular magnetic resonance (CMR) can detect necrosis and edema with high spatial resolution and it can quantify dyssynchrony by tagging techniques.

**Methods:**

Patients with a first AMI underwent percutaneous coronary interventions (PCI) of the infarct-related artery within 24 h of onset of chest pain. Within 5–7 days after the event and at 4 months, CMR was performed. The CMR protocol included the evaluation of intraventricular dyssynchrony by applying a novel 3D-tagging sequence to the left ventricle (LV) yielding the CURE index (circumferential uniformity ratio estimate; 1 = complete synchrony). On T_2_-weighted images, edema was measured as high-signal (>2 SD above remote tissue) along the LV mid-myocardial circumference on 3 short-axis images (% of circumference corresponding to the area-at-risk). In analogy, on late-gadolinium enhancement (LGE) images, necrosis was quantified manually as percentage of LV mid-myocardial circumference on 3 short-axis images. Necrosis was also quantified on LGE images covering the entire LV (expressed as %LV mass). Finally, salvaged myocardium was calculated as the area-at-risk minus necrosis (expressed as % of LV circumference).

**Results:**

After successful PCI (n = 22, 2 female, mean age: 57 ± 12y), peak troponin T was 20 ± 36ug/l and the LV ejection fraction on CMR was 41 ± 8%. Necrosis mass was 30 ± 10% and CURE was 0.91 ± 0.05. Edema was measured as 58 ± 14% of the LV circumference. In the acute phase, the extent of edema correlated with dyssynchrony (r^2^ = −0.63, p < 0.01), while extent of necrosis showed borderline correlation (r^2^ = −0.19, p = 0.05). PCI resulted in salvaged myocardium of 27 ± 14%. LV dyssynchrony (=CURE) decreased at 4 months from 0.91 ± 0.05 to 0.94 ± 0.03 (p < 0.004, paired *t*-test). At 4 months, edema was absent and scar %LV slightly shrunk to 23.7 ± 10.0% (p < 0.002 vs baseline). Regression of LV dyssynchrony during the 4 months follow-up period was predicted by both, the extent of edema and its necrosis component in the acute phase.

**Conclusions:**

In the acute phase of infarction, LV dyssynchrony is closely related to the extent of edema, while necrosis is a poor predictor of acute LV dyssynchrony. Conversely, regression of intraventricular LV dyssynchrony during infarct healing is predicted by the extent of necrosis in the acute phase.

## Background

Myocardial infarction (MI) causes formation of scar tissue and impairment of regional and global left ventricular (LV) function [1,2]. Typical characteristics of the altered motion pattern are reduced LV contraction [3,4] and post-systolic shortening in infarcted regions [5-7]. The assessment of LV dyssynchrony, which is a common finding in patients with MI [8], serves as a predictor for LV remodeling [9]. Therefore, measurement of mechanical LV function is an important factor to determine long-term prognosis and optimal treatment after MI.

Furthermore, assessment of LV regional contraction and dyssynchrony can give valuable insight with regard to cardiac resynchronization therapy (CRT) [10,11]. To date, CRT has proven successful in large patient populations as an adjunctive therapy for patients with drug-refractory heart failure and ventricular conduction delay. However, approximately 30% of these patients do not appear to benefit from CRT when applying current criteria (symptoms, LV-EF, and ECG findings) for CRT patient selection [10]. A modality that assesses regional LV mechanical function rather than LV electrical conduction fast and accurately could have great potential to improve patient selection for CRT [12]. Response rate for CRT has been reported to be lower in ischemic cardiomyopathy versus cardiomyopathies of other origin [13]. This could be related to the fact, that post-ischemic cardiomyopathy is likely to represent a dynamic process e.g. during infarct healing. However, little data exist on the development of dyssynchrony during post-infarction remodeling, and in particular, the influence of acute edema and necrosis on dyssynchrony is not known. Therefore, this study was undertaken to explore the evolution of dyssynchrony in relation to edema and necrosis/scar development after AMI and thus, to enhance our understanding of these processes to better evaluate in the future post-MI patients for CRT.

Cardiovascular magnetic resonance (CMR) with myocardial tagging [14] is a powerful method to assess deformation of the myocardium non-invasively. Three-dimensional (3D) tagging was introduced to allow the acquisition of complete 3D motion information covering the entire LV in only three breath-holds [15,16]. The method showed already comprehensive dyssynchrony quantification and high reproducibility in patients with left bundle branch block and acute MI [1]. In the current study, the 3D tagging method was used to measure LV contraction and dyssynchrony in patients after acute MI and at four months post-MI. It was reported in several previous studies that the amounts of scar tissue and healthy myocardium, as measured by late-gadolinium enhancement (LGE) imaging, correlate with important global LV function parameters such as ejection fraction and LV volumes [17,18]. Furthermore, it could be shown that the amount of LGE diminishes in MI patients from the acute to the chronic state [17,19]. CMR is also able to detect and quantify myocardial edema [20]. Therefore, in this study we used CMR to investigate changes in LV contraction and LV dyssynchrony in patients during the first four months after an acute MI. Specifically, it was hypothesized that both, necrosis and the size of the edema on T_2_-weighted images are responsible for dyssynchronic contractions of the LV in the acute phase. Secondly, the study was designed to investigate the influence of edema and necrosis in the acute phase on changes of LV dyssynchrony during infarct healing.

## Methods

### Study population

The study protocol was approved by the local Ethics Committee and all subjects gave written informed consent before study participation. Between November 2006 and August 2010, 22 patients (20 male/2 female, age = 56.9 ± 12.4 years) were examined at baseline 7.5 ± 4.1 days after first anterior MI and again at follow-up 4 months later (120.8 ± 21 days after MI). Ten patients participated in a previously published study [1]. Exclusion criteria for study participation were contraindications to CMR (typically incompatible metallic implants, claustrophobia). All recruited patients completed the study and no patients were lost to follow-up. Detailed characteristics and medications of all patients are given in Table 1, respectively.

**Table 1:** Patient Characteristics and Medications

	**Baseline (n=22)**	**Follow-up (n=22)**
**Gender, n (%)**		
**Male**	20 (91%)	
**Female**	2 (9%)	
**Age [years]**	56.9 (±12.4)	
**Body mass index [kg/m**^**2**^**]**	29.0 (±5.8)	
**Risk factors, n [%]**		
**Diabetes mellitus**	11 (50%)	
**Hypertension**	12 (55%)	
**Hyperlipidemia**	9 (41%)	
**Family history of CAD**	5 (23%)	
**Current smoker**	7 (32%)	
**History, n [%]**		
**Previous coronary angioplasty**	0 (0%)	
**Previous MI**	0 (0%)	
**Cardiac biomarkers**		
**Peak CK [U/l]**	4113.6 (±2339.7)	
**Peak CK MB [U/l]**	432.1 (±413.7)	
**Peak Troponin T [μg/l]**	20.3 (±35.7)	
**Coronary artery disease**		
**Number of obstructed vessels**		
**Infarct related vessel**†**, n [%]**		
**LAD**	22 (100%)	
**RCX**	0 (0%)	
**RCA**	0 (0%)	
**Acute coronary syndroms, n [%]**		
**STEMI**	22 (100%)	
**Medications, n [%]**		
**Aspirin**	2 (9%)	22 (100%)
**Beta-blocker**	0 (0%)	15 (68%)
**ACE inhibitor**	1 (5%)	18 (82%)
**Statin**	0 (0%)	22 (100%)
**Calcium channel blocker**	0 (0%)	0 (0%)
**Sartane**	0 (0%)	5 (23%)
**Diuretic**	0 (0%)	3 (14%)
**Clopidogrel**	0 (0%)	22 (100%)
**Oral anticoagulant**	0 (0%)	2 (9%)

Demographic characteristics, cardiac risk factors, history, cardiac biomarkers and coronary artery disease characteristics of all patients (n=22) included in the analysis are given.

MI = myocardial infarction; CAD = coronary artery disease; CK = creatine kinase; CK MB = creatine kinase isonenzyme MB; LAD = left anterior descending coronary artery; RCX = right circumflex coronary artery; RCA = right coronary artery; STEMI = ST-segment elevation myocardial infarction; NSTEMI = non ST-segment elevation myocardial infarction. A significant coronary stenosis was defined as ≥50% stenosis (percent luminar diameter narrowing). All data collected during the first examination. †Based on core laboratory analysis of the X-ray coronary angiograms. Medications of all patients with acute myocardial infarction (n=22) are listed for time of admission and at the time of follow-up. ACE = angiotensin-converting enzyme.

### Data acquisition

All measurements were performed on a 1.5 T MRI scanner (Achieva, Philips Healthcare, Best, The Netherlands). All acquisitions were performed during end-inspiration breath-hold periods. After acquiring survey images and reference data for sensitivity encoding (SENSE) [21] LV short-axis and long-axis images were acquired in order to plan subsequent scans. To assess global LV function, short-axis images covering the entire LV were then acquired in all patients using a steady state fee precession (SSFP) sequence (cardiac phases = 25, temporal resolution = 45 ms, slice thickness = 8 mm, 13–16 contiguous slices).

For the 3D tagging data acquisition an accelerated acquisition scheme was applied [1,16]. Imaging parameters were as follows: FOV = 108x108x108 mm^3^, matrix size = 28x14x16, receiver bandwidth = 220 Hz/pixel, number of profiles per EPI-segment = 7, number of excitations per heart phase = 4, echo time = 3.3 ms.

Edema imaging was performed with a standard T2-weighted (T2w) double-inversion black-blood spin-echo sequence (FOV = 350 × 350 mm^2^, pixel size = 1.4 × 1.4 mm^2^, repetition time = 2 R to R intervals, echo time = 100 ms, slice thickness = 8 mm) in 3 short-axis slices (basal, midventricular, and apical).

LGE short-axis images were acquired in all patients 15 minutes after administration of a bolus of 0.25 mmol/kg body weight of Gadobutrolum (Gadovist, Bayer Schering Pharma, Germany). An inversion recovery segmented gradient echo sequence was employed with an inversion time set to null normal myocardium (FOV = 350 × 350 mm^2^, pixel size = 1.5 × 1.5 mm^2^, repetition time = 7.4 ms, echo time = 4.4 ms, slice thickness = 8 mm, no slice gap). All MR data, and specifically the T2-weighted and LGE data, were acquired using a cardiac surface coil.

### Data analysis

The 3D tagging data were post-processed with HARP [22] using an extended software tool originally designed for the analysis of two-dimensional tagging data (TagTrack, GyroTools Ltd., Zurich, Switzerland). Eight to eleven short-axis midwall contours, consisting of multiple landmark points arranged in steps of about 5˚, were defined on different slices between base and apex. Midwall circumferential shortening (*csh* in %) was defined as the length change of a contour relative to the original length at end-diastole (first heart phase). For a detailed analysis of *csh* in 48–66 segments of each LV, each midwall contour was divided into sectors of 60˚ starting at the anterior epicardial junction of the right and the left ventricle on the equatorial level and numbered consecutively from S1 to S6 in clockwise direction as viewed from the apex. The time to maximum *csh* (T_max_) was automatically determined for each segment. Segments in which T_max_ was detected in the first three or in the last acquired time frame where inspected by an observer and corrected manually if necessary. As a measure for mechanical LV dyssynchrony the coefficient of variation in *csh* (CV_*csh*_*in %*) at end-systole, as well as the circumferential uniformity ratio estimate (CURE)-index were calculated [23,24]. CURE ranged from 0 (pure dyssynchrony) to 1 (synchronous). End-systole was defined in all patients as the moment of aortic valve closure assessed from SSFP images.

**Figure 1:**
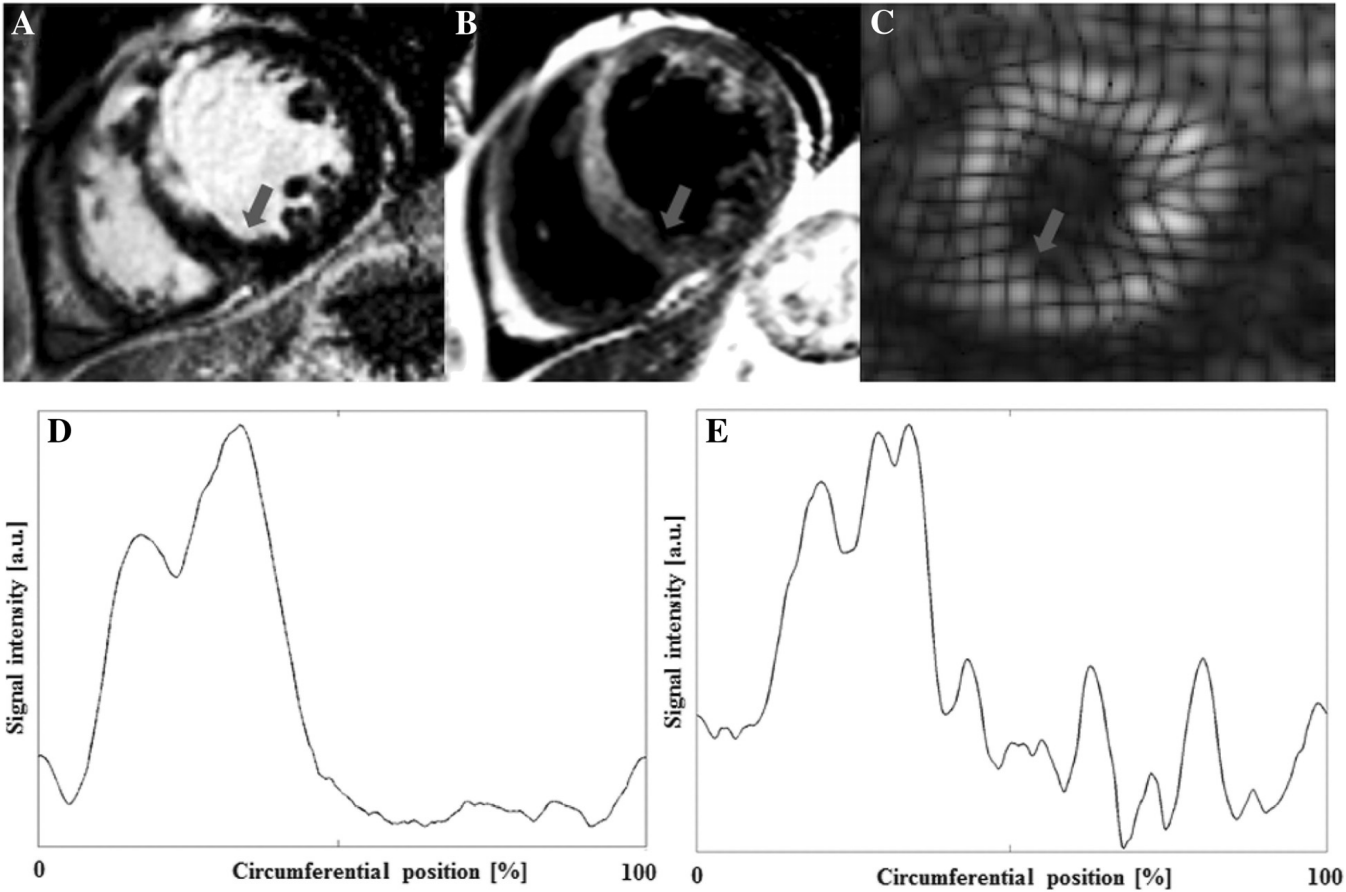
**Patient example (A) shows acute myocardial infarction (MI) on late gadolinium enhancement images and (B) corresponding edema in the anterior/anteroseptal segments on T2-weighted (T2) images and (C) shows corresponding 3D tagging at endsystole (grey arrows = inferior junction of the right ventricle (RV) with the interventricular septum represents the beginning of circumferential postion).** (**D**) represents circumferential signal intensities on late gadolinium enhancement images and (**E**) on T2-weighted images.

Data sets were post-processed using an in-house software (GTVolume, GyroTools Ltd., Zurich, Switzerland). For calculations of LV volumes and ejection fraction, endo- and epicardial borders were delineated manually on all acquired SSFP slices. On T_2_-weighted images, edema was measured as high-signal (>2 SD above remote tissue) along the LV mid-myocardial circumference on 3 short-axis images distributed equally along the LV long axis and was expressed as percentage of LV circumference. This evaluation along the LV mid-myocardial centreline was chosen to avoid the subendocardial region where tissue signals can be difficult to distinguish from signal of stagnant blood of the LV cavity. In analogy, on LGE images, necrosis was quantified manually as percentage of LV mid-myocardial circumference on 3 short-axis images. For this analysis 3 short-axis LGE images were selected from the stack of LGE images according anatomical landmarks that matched those of the T_2_-weighted images. The salvaged myocardium was calculated as area-at-risk (%LV circumference) minus necrosis (%LV circumference) and is expressed as %LV circumference (Figure 1). The total amount of necrosis and fibrosis was also quantified on LGE images covering the entire LV by summing of LGE tissue of all LV slices (expressed as %LV mass) in the acute and follow-up studies at 4 months.

### Statistics

Statistical analysis was performed by using the SPSS software package release 15.0.1 (Chicago, IL, USA) and MedCalc (Mariakerke, Belgium). Mean values + SD were calculated for all groups. The paired Student’s *t*-test or Wilcoxon test and independent samples *t*-test or Mann–Whitney test were used to test for differences within and between groups. All tests were two-tailed. The Kolmogorov–Smirnov test was used to test for normality. P-values less than 0.05 were considered statistically significant. The study was powered for the CURE index determined by 3D tagging CMR. A previous study [1] yielded a difference in CURE of 0.08 between controls and patients with old MIs assessed by CMR. Assuming that in the acute phase edema would decrease CURE by another 50% of this difference (= expected difference of 0.04 in CURE in the acute vs chronic MI), and considering a SD of 0.03 (intra-observer difference in (1) for CURE), a sample size of n = 18 (α = 0.05; power 80%) would be needed. A larger patient group was recruited to account for possible dropouts and a potentially smaller difference of the CURE index at 4 month follow-up, which was not known a priori.

## Results

Table 1 summarizes patient characteristics and medication. The mean age was 57 years and the majority of patients were male. All patients had an acute ST-segment elevation MI which was treated by PCI. The infarct-related artery was the left anterior descending coronary artery in all patients.

**Figure 2:**
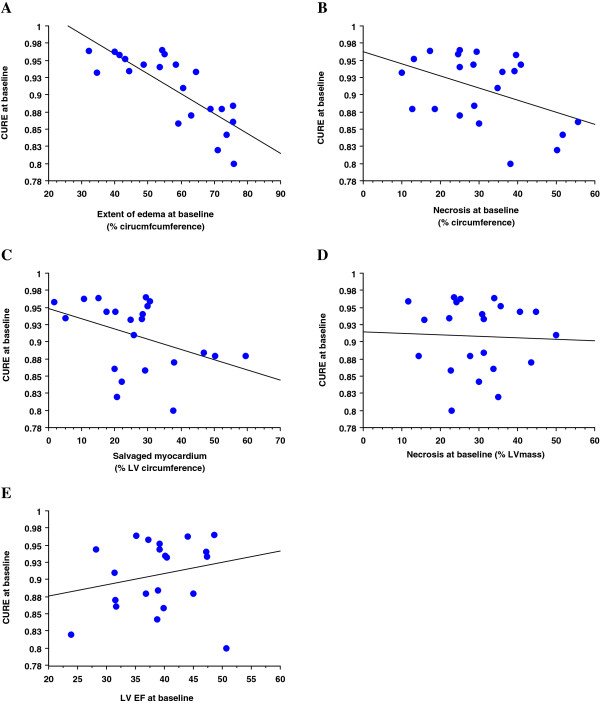
Predictors of LV dyssynchrony in the acute phase of MI.

Predictor of LV dyssynchrony in the acute phase of MI:

Figure 2 shows that in the acute phase of MI the circumferential extent of edema strongly correlated with dyssynchrony (Figure 2a, r^2^ = 0.63, p < 0.0001), while the circumferential extent of necrosis showed borderline correlation only (Figure 2b, r^2^ = 0.19, p = 0.05) as did the circumferential extent of salvaged myocardium (Figure 2c, r^2^ = 0.165, p = 0.06). Necrosis mass (Figure 2d, r^2^ = 0.02, p = 0.9) and LV ejection fraction (Figure 2e, r^2^ = −0.04, p = 0.4) did not correlate with dyssynchrony in the acute phase of MI.

Predictors of LV remodelling and LV dyssynchrony during healing of MI

**Figure 3:**
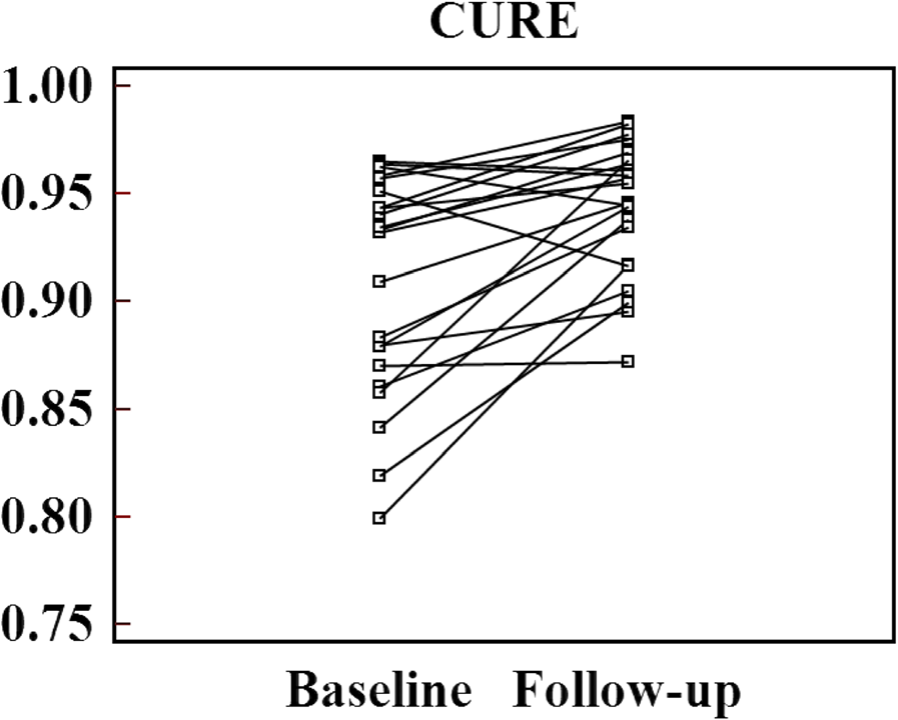
**Change of LV dyssynchrony at 4 months post-acute MI.** Lines track individual patient values at baseline to follow-up.

**Figure 4:**
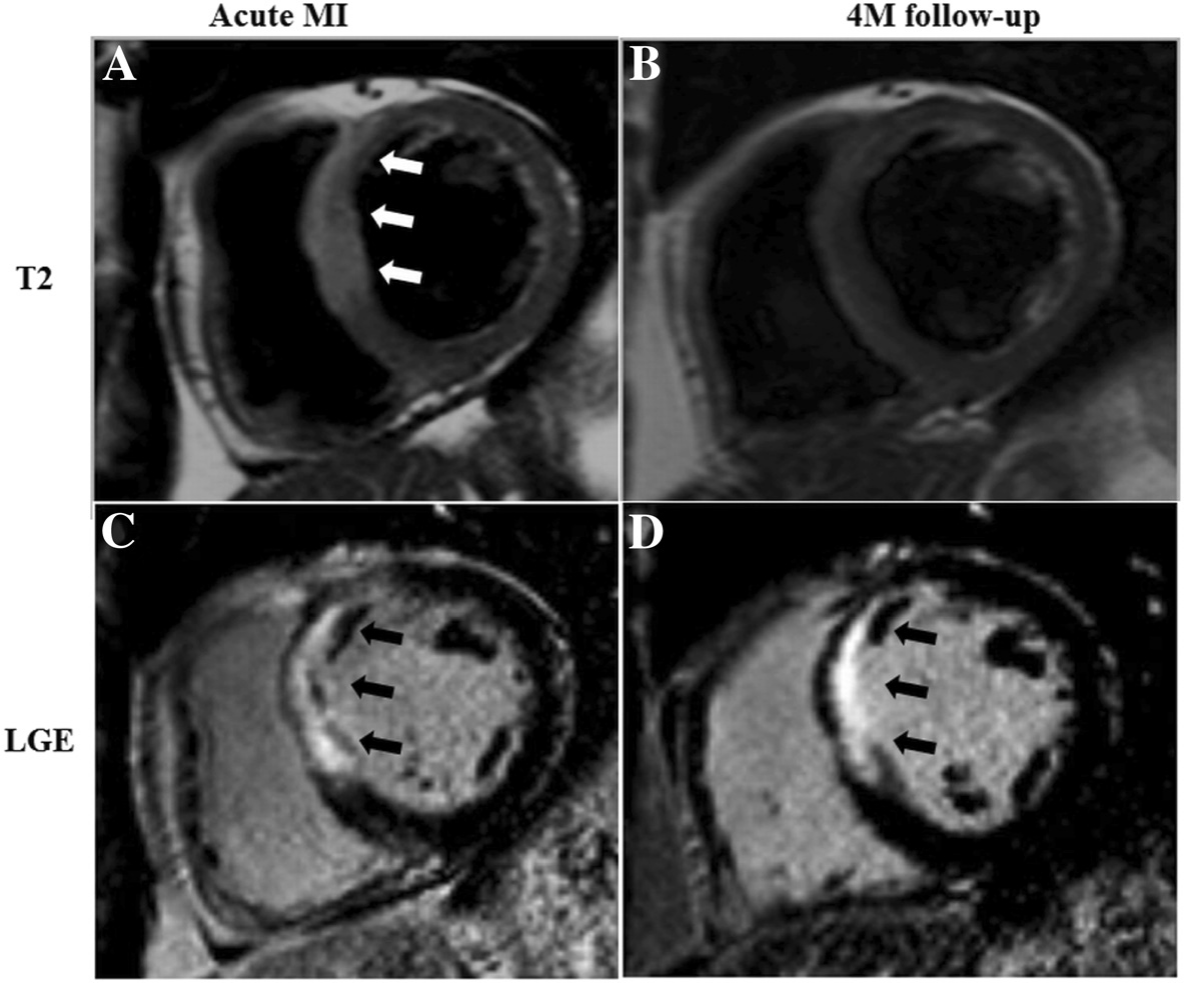
**Patient example with anterior myocardial infarction (MI).** Top row shows edema in the anterior/anteroseptal segments (white arrows) on T2-weighted (T2) short-axis images (equatorial slice) after acute MI (**A**) and absent edema at 4 months (**B**). Bottom row shows corresponding late gadolinium enhancement (LGE) images with scar formation in the anterior/anteroseptal segments (black arrows) after acute MI (**C**) and at 4 months (**D**).

A 4 months follow-up was available for all patients. The main study results are compiled in Table 2. Both, LVEDV and LVESV slightly increased, while LV ejection did not change significantly. LV scar shrunk slightly from 29.6% to 23.7% of LV mass at 4 months. When using LVEDV/LVmass as a measure of LV remodelling, an increase of this index occurred during the follow-up period (Table 2). At 4 months post-acute MI, intraventricular LV dyssynchrony was significantly reduced vs baseline (Figure 3; p = 0.004). In the 22 patients, no LV edema was detected at 4 months (see example in Figure 4).

**Table 2:** Study results

	**Baseline (n=22)**	**Follow-up (n=22)**	**P-value**
**Days after AMI**	7.5±4.1	120.8±20.6	-
**Heart rate (bpm)**	76±18	68±6	0.006
**LVEDD [mm]**	51.0±7.7	54.9±7.6	0.0005
**LVEDV [ml]**	156.8±50.5	177.9±66.4	0.01
**LVESV [ml]**	96.7±39.2	113.0±50.8	0.005
**LVEF [%]**	40.5±8.2	38.5±8.6	Ns
LV mass	133±36	121±30	0.02
LVEDV/LVmass	1.19±0.32	1.45±0.36	0.0004
**LV-scar (% LV mass)**	29.6±9.9	23.7±10.0	0.002
**% circumference Edema**	57.5±14.0	-	-
**% circumference Scar**	30.6±12.6	-	-
***csh*(AVC) mean [%]**	10.7±2.6	12.4±2.3	0.002
**T**_**max**_**mean [ms]**	345.7±34.5	390.8±35.4	<0.0001
**T**_**max**_**SD [ms]**	82.2±14.5	79.8±9.5	ns
**CURE**	0.91±0.05	0.94±0.03	0.004
**QRS width (ms)**	91.1±8.7	89.2±8.6	ns

Patients measured immediately after acute anterior myocardial infarction (baseline, n=22) and patients measured again four months later (follow-up, n=22) different studied parameters are listed: Left ventricular end-diastolic diameter (LVEDD), left ventricular end-diastolic volume (LVEDV), left ventricular end-systolic volume (LVESV), left ventricular ejection fraction (LVEF), scar mass, mean circumferential shortening (*csh*) over all segments of the left ventricle at aortic valve closure (AVC), mean and SD of time to maximum *csh* (T_max_) over all segments, the circumferential uniformity ratio estimate (CURE). All values are indicated as mean ± SD.

**Figure 5:**
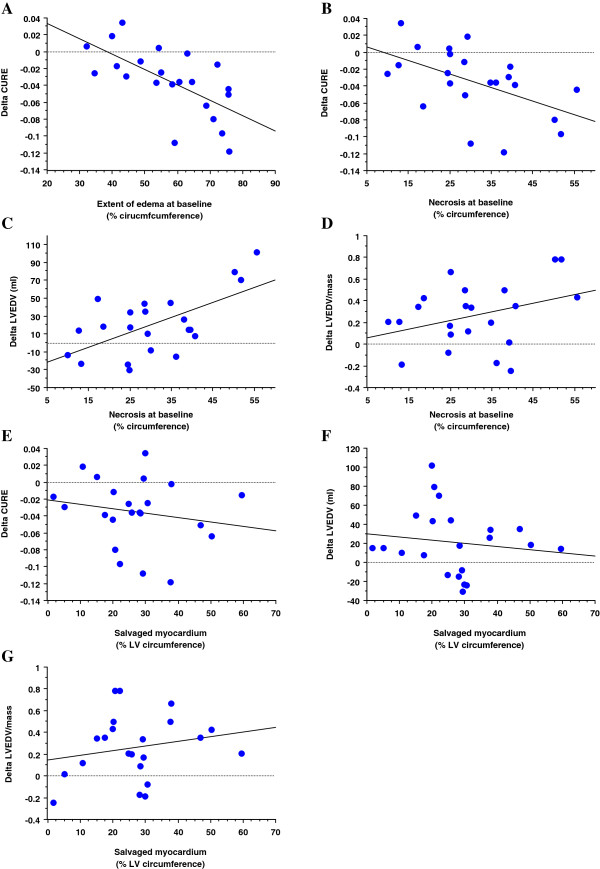
Predictors of LV remodelling and regression of dyssychrony during infarct healing.

Best predictor for regression of LV dyssynchrony during the 4 months follow-up period (expressed as ΔCURE) was the extent of edema in the acute phase (Figure 5 a). This correlation with edema extent was explained by its necrosis component (=extent of necrosis) which also correlated with regression of dyssynchrony (Figure 5b), while the portion of viable tissue in the edema territory (= salvaged myocardium) did not correlate with the regression of dyssynchrony (Figure 5e, r^2^ = 0.034, p = 0.41). Thus, the correlation between necrosis and regression of dyssynchrony indicates that LV dyssynchrony persisted in ventricles with smaller necrosis and improved in ventricles with larger necrosis. Extent of necrosis in the acute phase was also positively correlated with an increase in LVEDV (expressed as ΔLVEDV, Figure 5c) and the extent of necrosis showed borderline correlation with adverse LV remodelling (ΔLVEDV/LVmass, Figure 5d). Conversely, the extent of edema in viable tissue (=salvaged myocardium) did not correlate with ΔCURE, ΔLVEDV, nor ΔLVEDV/LVmass (Figure 5 e-g).

## Discussion

### Mechanisms of dyssynchrony in the acute phase of MI

MR tagging techniques [7] well as other modalities [25,26] could successfully demonstrate and quantify dyssynchrony in patients with chronic MIs. In this chronic stage, scar tissue is the substrate causing dyssynchrony, most likely by the mechanism of post-systolic shortening, by which the energy stored in scar tissue during contraction is released during early diastole [7]. In the present study, dyssynchrony was also observed in the setting of acute MI. The amount of hyperintense tissue on T2-w images correlated with the degree of dyssynchrony indicating that the underlying substrate for dyssynchrony in the acute phase is tissue edema, composed of both, viable edemateous tissue as well as the central necrotic tissue. The mass of necrotic tissue alone did not correlate significantly with the degree of dyssynchrony in the acute phase of MI. Thus, measurements that consider both, viable and non-viable edematous tissue best explain intraventricular LV dyssynchrony in the acute stage. This finding also suggests that dyssynchrony in the acute phase of MI may represent at least in part a transitory phenomenon.

### Mechanisms of regression of dyssynchrony during healing of acute MI

As was hypothesized, this study could demonstrate a regression of LV dyssynchrony during healing of acute MI (Table 2). Healing of acute MI was also associated in this patient population with an increase in LV end-diastolic and end-systolic volumes, as well as with an increase in LVEDV/LV mass, a measure of adverse remodelling. At 4 months scar tissue did shrink slightly as demonstrated earlier [27]. The reduction of dyssynchrony during the healing period was correlated with the amount of edematous tissue measured in the acute phase of MI (Figure 5a). As tissue edema can build up in necrotic as well as in viable myocardium, further analysis sought to investigate, which edema component is best predictive of regression of dyssynchrony. Interestingly, necrosis in the acute phase of MI inversely correlated with regression of dyssynchrony, thus indicating, that dyssynchrony tends to persist in ventricles with smaller necrotic mass, while dyssynchrony regresses in ventricles with larger necrosis. This might be explained by the fact, that in ventricles with very large scars, no sufficient contracting myocardium is left to cause intraventricular unloading and thus, post-systolic shortening. Extent of necrosis in the acute phase was positively correlated with LV dilation during infarct healing and a borderline correlation was observed for adverse LV remodelling during healing. Conversely, the edema mass in viable tissue during the acute phase of MI was not predictive for changes in LV dyssynchrony during healing, thus indicating, that salvaged myocardium (=edema in viable myocardium) is associated with dyssynchrony in the acute MI (Figure 2c), while it is not predictive for regression of dyssynchrony during healing (Figure 5e).

### Implications of the dynamic nature of dyssynchrony during infarct healing

From a practical point of view the transient nature of the intraventricular dyssynchrony during the acute phase of MI may suggest, that the indication for a CRT should be made with caution during the acute phase and a re-evaluation of dyssynchrony in the chronic phase is recommended when remodelling after MI has occurred. Also, in studies aimed to evaluate responder rates to CRT in patients after AMI, the stage of LV remodeling and the presence or absence of edema in the LV myocardium should be taken into account to enable a comprehensive assessment of the benefit of CRT in this patient population.

### Limitations

The extent of tissue edema was measured in 3 short-axis levels only, while tagging and LGE data were acquired over the entire LV. These 3 levels for edema assessment were chosen as the examination time could not be expanded deliberately since cooperation in acute MI patients is often decreasing during long examinations. A fully 3D edema quantification given as percentage of LV mass (and not as percentage of LV circumference) might have yielded tighter correlations with dyssynchrony. As mentioned in the methods section, for edema quantification, the extent of hyperintense tissue along the circumferential midline of each slice was calculated to avoid problems in discriminating edema tissue with high signal from high signal of stagnant blood in the LV cavity. As the edema distribution was typically transmural, this approach might not have affected substantially the correlations found between edema and dyssynchrony. Having these limitations in mind, we included in this study anterior infarcts only in order to study rather large infarcts. Thus, the findings of this study should not be extrapolated to smaller lateral or inferior infarcts.

Application of the accelerated 3D tagging acquisition technique allows assessing detailed 3D motion patterns of the entire LV in only three breath-holds. While the spatial resolution of the final motion parameters is relatively low (voxel size = 3.9 x 7.7 x 7.7 mm^3^) with respect to current imaging standards, regional motion changes typically occur on a larger scale in the vast majority of cardiac diseases including coronary artery disease and left bundle branch block. Hence, the achieved spatial resolution appears sufficient for most applications.

As a consequence of the rather small sample size, we cannot exclude that some correlations could be missed (or might be underestimated), however, the positive correlations found are not affected by this fact and show the importance of edema in the viable tissue to cause dyssynchrony. Furthermore, we tried to compensate for a small sample size by selecting a well defined patient population and by applying a strictly standardized imaging protocol.

## Conclusions

During infarct healing after revascularisation of acute anterior MI, intraventricular LV dyssynchrony regresses. In the acute phase of infarction, LV dyssynchrony is closely related to the extent of edema, while necrosis alone does not explain dyssynchrony in the acute phase. Conversely, regression of dyssynchrony during infarct healing is best predicted by the extent of necrosis in the acute phase.

From a practical point of view, the indication for CRT in post-infarct patients should consider the potential for improvement of dyssynchrony during infarct healing which is related to necrosis mass in the acute MI. Also, in studies assessing responder rates of CRT in ischemic cardiomyopathy the stage of infarct healing and the presence or absence of tissue edema should be taken into account as these factors have been shown to modify dyssynchrony severity.

## Abbreviations

CMR, Cardiovascular magnetic resonance; AMI, Acute myocardial infarction; CAD, Coronary artery disease; LGE, Late-gadolinium enhancement; FOV, Field of view; CURE, Circumferential uniformity ratio estimate; LV, Left ventricle; BMI, Body mass index; PCI, Percutaneous coronary intervention; CRT, Cardiac Resynchronization Therapy; STEMI, ST-segment elevation myocardial infarction; NSTEMI, Non ST-segment elevation myocardial infarction.

## Competing interests

The authors declare that they have no competing interest.

## Authors' contributions

RM: study design, data acquisition, CMR image analysis, statistical analysis, and manuscript drafting. SK: data acquisition, CMR image analysis, and manuscript drafting. AKR: CMR image analysis, literature research, and manuscript drafting. CS: CMR image analysis and manuscript drafting. PB: study design, literature research, and manuscript drafting. JS: study design, statistical analysis, revision of the manuscript, and guarantator of integrity of entire study. All authors have made revisions to the manuscript and have read and approved the final version.
